# Protective effect of adenosine triphosphate against cisplatin-induced necrotic and degenerative oral mucositis in rats

**DOI:** 10.1590/1678-7757-2025-0007

**Published:** 2025-03-24

**Authors:** Ismail Salcan, Muhammed Dilber, Zeynep Suleyman, Nurinisa Yucel, Sara Salcan, Sefa Kesan, Gulce Naz Yazici, Fatih Celik, Merve Koseturk, Nurdan Alcan Alp, Halis Suleyman

**Affiliations:** 1 Erzincan Binali Yildirim University Faculty of Medicine Department of Ear, Nose, Throat Diseases Erzincan Turkey Erzincan Binali Yildirim University, Faculty of Medicine, Department of Ear, Nose, Throat Diseases, Erzincan, Turkey.; 2 Independent researcher Istanbul Turkey Independent researcher, Istanbul, Turkey.; 3 Erzincan Binali Yildirim University Faculty of Health Sciences Department of Internal Medicine Nursing Erzincan Turkey Erzincan Binali Yildirim University, Faculty of Health Sciences, Department of Internal Medicine Nursing, Erzincan, Turkey.; 4 Erzincan Binali Yildirim University Vocational School of Health Services Erzincan Turkey Erzincan Binali Yildirim University, Vocational School of Health Services, Pharmacy Services Program, Erzincan, Turkey.; 5 Erzincan Binali Yildirim University Faculty of Medicine Department of Public Health Erzincan Turkey Erzincan Binali Yildirim University, Faculty of Medicine, Department of Public Health, Erzincan, Turkey.; 6 Kozlu State Hospital Department of Ear, Nose, Throat Diseases Batman Turkey Kozlu State Hospital, Department of Ear, Nose, Throat Diseases, Batman, Turkey.; 7 Erzincan Binali Yildirim University Faculty of Medicine Department of Histology and Embryology Erzincan Turkey Erzincan Binali Yildirim University, Faculty of Medicine, Department of Histology and Embryology, 24100, Erzincan, Turkey.; 8 Erzincan Binali Yildirim University Oral and Dental Health Education and Research Hospital Erzincan Turkey Erzincan Binali Yildirim University, Oral and Dental Health Education and Research Hospital, Erzincan, Turkey.; 9 Erzincan Binali Yildirim University Faculty of Dentistry Department of Endodontics Erzincan Turkey Erzincan Binali Yildirim University, Faculty of Dentistry, Department of Endodontics, Erzincan, Turkey.; 10 Erzincan Binali Yildirim University Faculty of Medicine Department of Pharmacology Erzincan Turkey Erzincan Binali Yildirim University, Faculty of Medicine, Department of Pharmacology, 24100, Erzincan, Turkey.; 11 Erzincan Binali Yildirim University Faculty of Medicine Department of Medical Biochemistry Erzincan Turkey Erzincan Binali Yildirim University, Faculty of Medicine, Department of Medical Biochemistry, 24100, Erzincan, Turkey.

**Keywords:** Adenosine triphosphate, Cisplatin, Tongue, Rat, Oxidative injury

## Abstract

Inflammation, oxidative damage, and adenosine triphosphate (ATP) depletion play a role in the pathogenesis of cisplatin (CIS)-induced oral mucositis.

**Objective:**

The purpose of this research is to examine the impact of ATP against potential oral mucositis development in cisplatin-treated rats. Methodology All rats were randomly assigned to four groups, namely healthy control group (HG), ATP group (ATPG), Cisplatin group (CISG), and ATP + Cisplatin group (ATCS). Firstly, ATP 4 mg/kg was administered via intraperitoneal injection (IP) to both ATPG and ATCS groups. The same volume of normal saline was injected into HG and CISG groups. After 1 h, cisplatin 5 mg/kg was administered via IP to CISG and ATCS groups. The drugs were taken 1x1 for 7 d. Later, tongue tissues were collected from all groups. Biochemical, macroscopic, and histopathological examinations were performed on all tissues.

**Results:**

ATP inhibited cisplatin-induced oxidative damage and pro-inflammatory cytokines levels in tongue tissue. In the CIS group, a significant number of distinct sulcus formations were found in the apex and corpus, as well as a few ulcer foci in the corpus, significant papilla loss, and bleeding. Meanwhile, in the ATP group, a similar appearance to healthy tissue was observed. Histopathologically, it was determined that in cisplatin-aggravated tongue tissue damage, filiform papillae decreased when ATP was administered, and the arrangement and structures of the epithelium, blood capillaries, muscle groups, and adipose cell groups were normal.

**Conclusions:**

Oral mucositis caused by cisplatin is alleviated by ATP. These findings may be useful for developing new therapeutic approaches to prevent or treat mucositis, a side effect so severe that can lead to treatment discontinuation.

## Introduction

Cisplatin is a platinum-based chemotherapy agent with a wide range of indications. These include bladder, head and neck, lung, ovarian, testicular and germ cell carcinomas, lymphomas, and sarcomas.^1^ Its anticancer mechanism is associated with its capacity to cross-link with purine bases in DNA, causing DNA damage-induced apoptosis in cancer cells.^2^ However, its use is limited due to drug resistance, and many severe adverse effects, such as kidney disorders, allergic reactions, reduced immunity to infections, gastrointestinal diseases, bleeding, and hearing loss.^1^ Additionally, platinum-based drugs can require dose reductions of 25%–100% due to hepatotoxicity, ototoxicity, cardiotoxicity, pain, mucositis, and stomatitis.^3^ Mucositis is a widespread adverse impact of many chemotherapy drugs; however, it has been reported to be more severe in patients receiving cisplatin.^4^ Oral mucositis is a major adverse effect of chemotherapy that can negatively impact a patient's cancer treatment outcome.^5^ The pathogenesis of mucositis involves steps, such as damage to the mucosal membranes, the release of reactive oxygen species (ROS), and the stimulation of proinflammatory genes, including nuclear factor-κB (NF-κB), tumor necrosis factor-alpha (TNF-α), interleukin-6 (IL-6), and interleukin-1beta (IL-1β) by damaged mucosal cells.^6^ Oxidative stress increases mitochondrial electron transport leading to elevated H_2_O_2_ production, depletion of adenosine triphosphate (ATP), and ultimately cell death.^7^ A significant reduction in ATP levels and cell energy depletion is a critical factor in cisplatin-induced cytotoxicity.^8^ These findings suggest that ATP is essential for maintaining and preserving antioxidant activity in cells and tissues. This highlights the importance of ATP depletion and proinflammatory cytokine elevation in the pathogenesis of cisplatin-associated oral mucositis. ATP is a nucleoside triphosphate consisting of adenine, ribose sugar, and three phosphate groups.^9^ Reportedly, ATP participates in the synthesis of ROS-scavenging and detoxifying antioxidants.^10^ This suggests that in the treatment of oral mucositis associated with the use of cisplatin, ATP may be useful. No data are accessible on the effect of ATP against cisplatin-induced oral mucositis damage. The purpose of the present research is to test the impact of ATP against possible oral mucositis development in cisplatin-treated rats.

## Methodology

### Rats

In total, 24 male albino Wistar rats weighing 278–289g were used. All rats were obtained from the Medical Experimental Application and Research Centre of Erzincan Binali Yildirim University. Before the experiment, the animals were grouped (n = 6) and housed under laboratory conditions at an ambient temperature of 22±2°C, with a 12-h light/dark cycle and a humidity level of 35–65 %. The rats had access to standard pellet feed (Bayramoglu AS, Erzurum/Turkey) and water ad libitum. The procedures were approved by the Local Animal Ethics Committee (Date:25.11.2024, Meeting No: 11/51).

### Chemicals

The ATP was provided by Zdorove Narodu (Ukraine), Cisplatin (CDDP) vials (50 mg/100 ml; Cisplatin) were obtained from Ebewe Liba (Türkiye), and thiopental sodium was supplied by I.E. Ulagay (Turkey).

### Experimental groups

The experimental animals were grouped into four groups (N=6),

1. HG: Healthy control group
2. ATPG: Administered ATP 4 mg/kg
3. CISG: Administered Cisplatin 5 mg/kg
4. ATCS: Administered ATP 4 mg/kg + Cisplatin 5 mg/kg

### Experimental procedure

Rats in the ATPG and ATCS groups underwent intraperitoneal injection (IP) of 4 mg/kg ATP.^11^ The other two groups, HG and CISG, received the same volume of normal saline (%0.9 NaCl) IP as the solvent. After 1 h, the ATCS and CISG groups underwent IP of 5 mg/kg Cisplatin.^12^ The drugs were taken 1x1 for 7 d. Then, all rats were euthanized with high-dose anesthesia (50 mg/kg thiopental sodium), and their tongue tissues were collected. The excised tongue tissues were analyzed for malondialdehyde (MDA), total glutathione (tGSH), superoxide dismutase (SOD), catalase (CAT), tumor necrosis factor-alpha (TNF-α), interleukin-6 (IL-6), and interleukin-1beta (IL-1β) levels. The tissues were examined macroscopically and histopathologically. Subsequently, results were compared and evaluated.

### Biochemical analysis

#### Tissue MDA, tGSH, SOD, and CAT analysis

Tissue MDA, GSH, and SOD levels were surveyed using rat ELISA kits and each assay was conducted following the instructions of the manufacturer (CAT no: 706002, 703002, and 10009055, Cayman Chemical Company, respectively). CAT levels were determined following the method proposed by Goth.^13^

#### Tissue TNF-α, IL-1β, and IL-6 analysis

The tissues were weighed and washed with physiological saline, then powdered in liquid nitrogen and homogenized. The levels of tumor necrosis factor α (TNF-α; ng/tissue), Interleukin 1 beta (IL-1beta; pg/tissue), and Interleukin 6 (IL-6; ng/tissue) were determined using kits purchased from Eastbiopharm Co Ltd following manufacturer's instructions.

### Histopathological analysis

The tissue specimens were fixed with 10% formaldehyde. They were dehydrated via a series of alcohols after washing in running water. After clearing in xylene, the tissues were paraffin-embedded. Paraffin sections were stained with hematoxylin-eosin and examined by light microscopy (Olympus Inc., Tokyo, Japan). For semi-quantitative analysis, one central and five peripheral areas were selected from serial sections. Each sample was scored as: absent (0); mild (1); moderate (2); and severe damage (3). Tongue tissue damage was defined as the presence of "epithelial necrosis, muscle degeneration, PMNL infiltration, and edema." A pathologist blinded to the experimental groups performed the evaluation (Table 1).

**Table 1:** Histopathological scores

	Epithelial necrosis	Muscle degeneration	PMNL infiltration	Edema
Tongue tissue	0-1 +++	none -	none -	<10% -
	1-3 ++	1-5% +	1-3% +	10-30% +
	3-5 +	5-10% ++	3-5% ++	30-50% ++
	>5 -	>10% +++	>5% +++	>50% +++

### Statistical analysis

The results were stated as mean ± standard deviations (x ± SD). Shapiro Wilk test was employed to determine whether the groups were normally distributed for biochemical analysis. The significance of differences between groups was determined using the one-way analysis of variance (ANOVA) test, followed by the Tukey test for post-hoc. Histopathological analysis was performed by employing the Kruskal Wallis test. All statistical analyses were performed using the SPSS for Windows, 25.0 (Armonk, NY: IBM Corp.) and the GraphPad Prism 8. A *p*-value of <0.05 was defined as statistically significant.

## Results

### Biochemical findings

#### Oxidant/Antioxidant levels in tongue tissue

**Figure 1:**
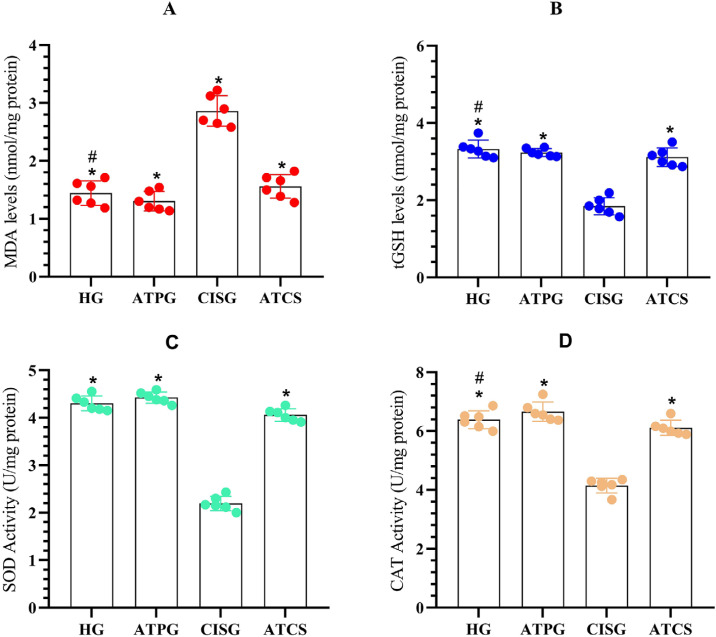
Oxidant and antioxidant levels in rat tongue tissue. Malondialdehyde (MDA), total glutathione (tGSH), superoxide dismutase (SOD), and catalase (CAT). Groups included Healthy control (HG), ATP-alone (ATPG), Cisplatin-alone (CISG), and ATP + Cisplatin (ATCS). p<0.05 was determined as significant (N=6). Superscript * indicates p<0.05 vs CISG; # indicates p<0.05 vs ATPG and ATCS groups.

As shown in Figure 1A, no statistically significant difference was found in MDA levels in the tongue tissues of the HG and ATPG groups. However, the MDA level in the tongue tissue of the CISG group, which was administered cisplatin alone, was significantly higher compared to the HG and ATPG groups.

In Figure 1B, tGSH levels in the tongue tissues of the HG and ATPG groups were almost similar and the difference in tGSH levels between these groups was not significant. Cisplatin administered alone decreased tGSH levels. The tGSH levels in the tongue tissues of the CISG group were significantly lower than those of the HG and ATP groups. While the MDA and tGSH levels in the ATCS group that administered ATP and cisplatin were found to be close to the levels in the HG and ATPG groups, the MDA and tGSH levels in the CIS group were estimated to be statistically significant.

As seen in Figure 1C-D, the SOD and CAT activities in the tongue tissues of animals administered cisplatin alone were significantly lower than those of the HG and ATPG groups. Moreover, ATP significantly prevented the decrease in SOD and CAT activities caused by cisplatin. However, no significant difference was found in the SOD and CAT activities between the HG and ATPG groups. SOD levels of animals in the ATCS group were found to differ significantly compared to the HG group, whereas CAT levels were found to show no significant differences. In addition, SOD and CAT levels in the ATCS group were estimated as compared to the CIS group and compared to the ATPG group.

### Pro-inflammatory cytokines levels in tongue tissue

**Figure 2:**
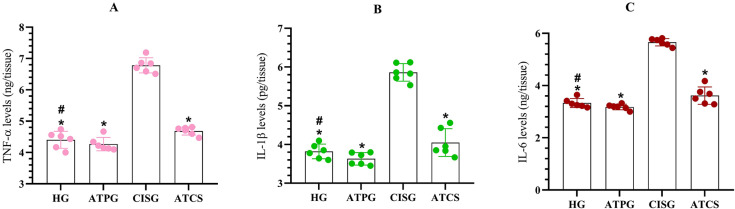
Pro-inflammatory cytokine levels in rat tongue tissue. Tumor necrosis factor-alpha (TNF-α), interleukin-1beta (IL-1β), and interleukin-6 (IL-6). Groups included Healthy control (HG), ATP-alone (ATPG), Cisplatin-alone (CISG), and ATP + Cisplatin (ATCS). p<0.05 was determined as significant (N=6). Superscript * indicates p<0.05 vs CISG; # indicates p<0.05 vs ATPG and ATCS groups.

As seen, cytokine levels of TNF-α, IL-1β, and IL-6 in the tongue tissues of the HG and ATPG groups were significantly lower than that of the CISG group. Moreover, the differences in cytokine levels between the HG and ATPG groups were not statistically significant. TNF-α, IL-1β, and IL-6 levels in the ATCS group were found to be significantly higher than those in the HG. In addition, proinflammatory cytokines levels in the ATCS group were significantly higher than those in the CIS and the ATPG group (Figure 2).

### Macroscopic findings of tongue tissue

**Figure 3:**
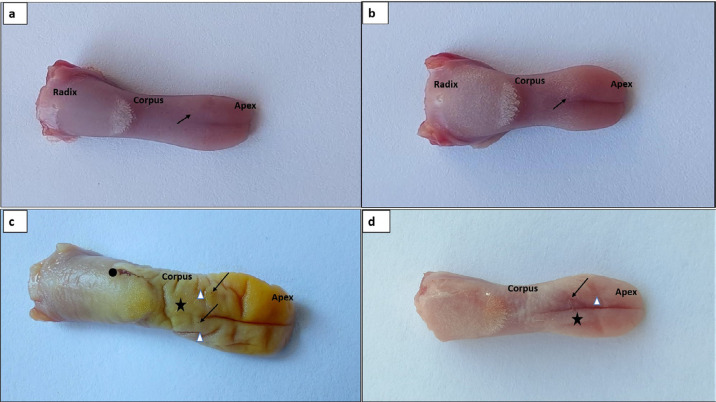
Macroscopic view of tongue tissue from the study groups. a. Healthy control group: normal apex, corpus, radix, epithelial layer, and papillae (arrow); b. ATPG group treated with ATP alone: normal apex, corpus, radix, epithelial layer, and papillae (arrow); c. CISG group treated with cisplatin alone marked: edema and abnormal coloration throughout the tongue tissue; formation of multiple distinct sulci in the apex and corpus (Δ), isolated ulcer foci in the corpus (arrow), notable loss of papillae (★), and hemorrhage (●); d. ATCS group treated with ATP + Cisplatin: mild edema and near-normal coloration throughout the tongue tissue; normal apex structure, a few mild sulcal formations in the corpus (Δ), isolated ulcer foci in the corpus (arrow), and mild loss of papillae (★)

As seen in Figure 3a, macroscopically normal apex, corpus, radix, epithelial layer, and papillae in the tongue tissue of the HG. The absence of histopathology was observed in the tongue tissue of the ATPG, which received ATP alone (Figure 3b). However, in the tongue tissue of the CISG group, which was administered cisplatin alone, marked edema (75%) and abnormal coloration were noted throughout the entire tissue. Numerous pronounced sulcus formations (16 pieces) were observed in the apex and corpus, with seven ulcer foci in the corpus, significant papilla loss (75%), and one large hemorrhage (Figure 3c). In the ATCS group, treated with ATP and cisplatin, mild edema (25%) and near-normal coloration of the tongue tissue were observed, with the apex appearing normal. In total, three sulcus formations and two ulcer foci were noted in the corpus. Additionally, mild loss of papillae (25%) was observed in the tongue tissue of this group; however, hemorrhage was undetected (Figure 3d). Table 2 presents the macroscopic findings.

**Table 2:** Resuls of macroscopic appearance

	Ulcer	Sulcus	Papillae	Hemorrhage	Edema
HG	0	0	0	0	0
ATPG	0	0	0	0	0
CISG	7	16	75%	1	75%
ATCS	2	3	25%	0	25%

HG; Healthy control group, ATPG: Adenosine triphosphate alone, CISG: Cisplatin alone, ATCS: ATP+ Cisplatin.

### Histopathological findings

**Figure 4:**
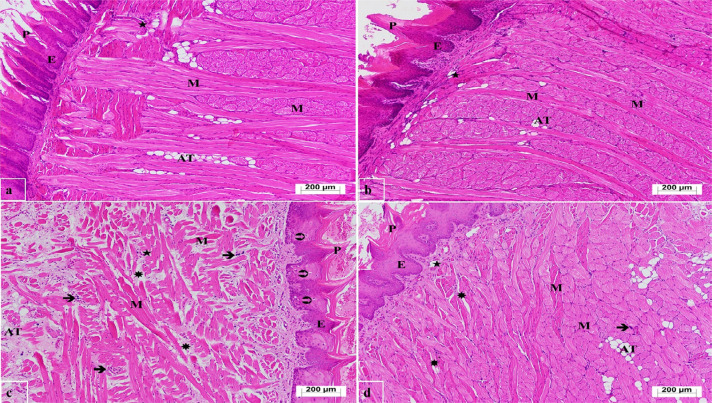
Histopathological appearance of tongue tissue in each group (H&E x 100). a. Healthy control group; **P**: filiform papilla, **E**: stratified squamous epithelium, **M**: muscle tissue, **AT**: adipose cell groups, ★: blood vessels, **b**. ATPG group; **P**: filiform papilla, **E**: stratified squamous epithelium, **M**: muscle tissue, **AT**: adipose cell groups, ★: blood vessels, **c**. CISG group; **P**: filiform papilla with reduced density, **E**: swollen cells in stratified squamous epithelium, ➲: necrotic cells in the epithelium, **M**: degenerated muscle groups, ✷: edematous areas, **AT**: degenerated and reduced adipose cell groups, ➔: widespread polymorphonuclear cell infiltration, ★: blood vessels, **d**. ATCS group; **P**: reduced number of filiform papillae, **E**: stratified squamous epithelium, **M**: muscle tissue, ✷: focal edematous areas, **AT**: adipose cell groups, ➔: polymorphonuclear cell infiltration,★: blood vessels.

As shown in Figure 4a, the tongue tissue sections from the HG group displayed a normal histological structure with a dense arrangement of filiform papillae, a surface covered by stratified squamous epithelium underlying muscle layers in all directions, and adipose cell clusters and blood capillaries scattered between the muscle layers. The morphological structure of the tongue tissue in the ATPG group, treated with ATP alone, exhibited a similar histological pattern to that of the HG group (Figure 4b). Conversely, examination of the tongue tissue from the CISG group, which received cisplatin, revealed a reduction in the density of filiform papillae compared with the HG group, swelling of the epithelial cells, and the presence of necrotic cells within the epithelium. It was observed that the normal arrangement of muscle groups was disrupted and degenerated, as well as intense edematous areas between the muscle groups, intense polymorphonuclear cell infiltration around the blood capillaries and throughout the tissue, and degenerated adipose cell groups along with decreased distribution (Figure 4c). In the ATCS group, treated with ATP and cisplatin, the tongue tissue showed a reduced number of filiform papillae, but the epithelium maintained a normal structure, similar to that of the control group. The arrangement and structure of the blood capillaries, muscle groups, and adipose cell clusters appeared normal. Mild edema persisted between the muscle layers, and polymorphonuclear cells were intermittently present throughout the tissue (Figure 4d). Table 3 presents the histopathological scoring.

**Table 3:** Histopathological damage grading

Findings	Groups							
	Median ± 95 % Confidence Interval							
	HG		ATPG		CISG		ATCS	
	Median	Lower bound	Median	Lower bound	Median	Lower bound	Median	Lower bound
		Upper bound		Upper bound		Upper bound		Upper bound
Epithelial necrosis	0	0	0	0	3*	2.5685	1**	0.3850
		0		0		2.8759		0.7261
Muscle degeneration	0	0	0	0	3*	2.6014	1**	0.8383
		0		0		2.8986		12.172
PMNL infiltration	0	0	0	0	3*	2.5364	1**	0.9410
		0		0		2.8525		1.4479
Edema	0	0	0	0	3*	2.4142	1**	1.1302
		0		0		2.7525		1.5921

0: absent; 1, mild damage; 2, moderate damage 3, severe damage.

* p<0.001 vs. HG, ATPG, and ATCS,

** p>0.05 vs. HG, and ATPG, HG; Healthy control group, ATPG: Adenosine triphosphate alone, CISG: Cisplatin alone, ATCS: ATP+ Cisplatin.

*Statistical analysis was performed by Kruskal Wallis test.

## Discussion

In this study, the impacts of ATP on cisplatin-induced oral mucositis, a common side effect in the care of various malignant solid tumors, were investigated macroscopically, biochemically, and histopathologically. The tongue tissue of rats was used to evaluate oral mucositis. The literature shows that, in addition to its therapeutic effects, cisplatin also causes many side effects, including oral mucositis.^14^ As described above, mucositis progresses by steps, including damage to the mucosal membranes, release of ROS, and stimulation of pro-inflammatory cytokine genes by damaged mucosal cells.^6^ The pathogenesis of cisplatin-induced oxidative stress has been linked to an increase in MDA levels, a ROS product, and a decrease in the levels of endogenous antioxidant biomarkers GSH, SOD, and CAT.^15,16^ Based on information from the current literature, MDA, tGSH, SOD, and CAT levels were measured in tongue tissue to investigate the potential pathogenesis of mucositis caused by cisplatin. As demonstrated by our results, the MDA levels in the tongue tissues of animals treated with cisplatin alone were significantly higher than those in the healthy, ATPG, and ATCS groups. Recent studies have shown that an increase in ROS and MDA levels leads to an exacerbation of oral mucositis.^17^ Cisplatin-induced increase in ROS levels promotes lipid peroxidation (LPO) and triggers the production of MDA, one of the end products of this process.^16^ Therefore, MDA is widely used as a biomarker to assess oxidative stress.^18^ Additionally, MDA levels in tissues are used as biomarkers in the detection of various diseases in both *in vivo* and *in vitro* studies.^19^ A monoclonal antibody, which acts as an endogenous agent against oxidative stress, specific to MDA has been developed to detect DNA damage in human oral mucosa.^18^ Numerous studies in the literature have linked cisplatin toxicity to elevated MDA levels.^16,20^

Nguyen, et al.^17^ (2022) explained that the decrease in ROS and MDA levels is followed by an increase in GSH and SOD, resulting in a decrease in the severity of oral mucositis. As mentioned above, the severity of cisplatin-induced oxidative stress is linked to a decrease in GSH, SOD, and CAT levels.^15,16^ These antioxidants, which were evaluated in the present study, neutralize and render ROS and their toxic products harmless and aid maintain tissue integrity.^21^ Our biochemical findings, in line with the literature, demonstrated that cisplatin increased oxidant levels while reducing antioxidant levels in the tongue tissue of rats. In summary, the interaction of antioxidants with ROS can lead to a decrease in oxidants and the consumption of antioxidants. Therefore, antioxidants are recommended in the treatment of oxidative stress. Basak, et al.^22^ (2021) also demonstrated the benefits of antioxidants in the treatment of cisplatin-induced mucositis.

Oral mucositis occurs as an inflammatory condition of the oral mucosa due to the cytotoxic effects of cisplatin.^6^ A study suggested that ROS is the underlying factor of oral mucositis.^23^ Another study argued that ROS are thought to directly trigger inflammatory events that exacerbate cellular damage.^24^ Mucositis involves processes, such as the stimulation of pro-inflammatory cytokine genes via mucosal cells after ROS release.^6^ During this process, there is an increased expression of pro-inflammatory cytokines such as NF-κB, TNF-α, IL-6, and IL-1β.^25^ In the present study, the concentrations of pro-inflammatory cytokines, such as TNF-α, IL-1β, and IL-6 were measured to demonstrate that oxidants trigger inflammatory events in oral mucositis. Consistent with the literature, in this study, the pro-inflammatory cytokine levels in the tongue tissues of cisplatin-treated rats were higher than those in the HG, ATPG, and ATCS groups. These findings suggest that cisplatin may induce oral mucositis by causing oxidative damage as well as by activating pro-inflammatory pathways.

In cisplatin cytotoxicity, a significant decrease in ATP levels and energy depletion of cells have been highlighted as critical factors.^8^ It has been reported that a reduction in ATP concentrations leads to oxidative stress and membrane LPO.^26^ The findings obtained in the present study suggest no meaningful difference in oxidant and antioxidant levels in the tongue tissues of healthy animals and those treated with only ATP. Moreover, ATP suppressed the increase in oxidant markers and the depletion of antioxidant molecule reserves in the tongue tissues of cisplatin-treated animals. Supporting this finding, Bouitbir, et al.^27^ (2019) reported that an increase in mitochondrial ROS production leads to a decrease in cellular ATP. In a study hypothesizing that mucositis is caused by ATP depletion, it was reported that administration of exogenous ATP alleviated mucositis development.^28^ Moreover, in the present study, ATP significantly inhibited the increase in pro-inflammatory cytokine levels in the tongue tissues of cisplatin-treated animals. ATP is converted into adenosine in cells and inhibits the production of pro-inflammatory cytokines by stimulating adenosine receptors.^29^ Moreover, it is necessary for the release of unconventionally secreted cytokines, such as IL-1β, paradoxically inhibits the release of conventionally secreted cytokines like TNF-α.^30–33^ In addition, ATP also causes an increase in IL-10 production.^34^ According to the findings obtained in the present study, ATP suppressed the increase in TNF-α, IL-1β, and IL-6 levels in the tongue tissues of cisplatin-treated animals. Based on our results, we believe that ATP may have reduced the increase in pro-inflammatory cytokines by suppressing oxidant production in the tongue tissues of the rats. These findings are consistent with literature evidence showing that ROS triggers inflammatory events that exacerbate cellular damage.^24^

Chemotherapy causes significant morphometric and microscopic changes in the oral mucosa.^35^ In the tongue tissues of the ATPG, no macroscopic damage was observed. However, in the tongue tissues of animals treated with cisplatin alone, significant damage was evident. Additionally, ATP alleviated the edema, loss of papillae, occasional ulcer foci, and hemorrhage in the tongue tissues induced by cisplatin. In a recent study, it was stated that an anticancer drug macroscopically caused similar damage in the animal oral mucosa.^36^ These macroscopic findings are consistent with our biochemical and histopathological results. There are several studies in the literature in which cisplatin-induced oral mucositis was examined macroscopically and histopathologically. These studies have demonstrated a significant increase in confluent ulceration or pseudomembrane formation along with bleeding in the tongue tissues of rats on the 2^nd^ and 3^rd^ days of cisplatin administration.^37^ In a different study, Egilmez, et al.^14^ (2020) observed moderate vasodilatation, epithelial hydropic degeneration, presence of hemorrhagic, edema, and ulcer areas in the mucosal tissues of cisplatin-treated rats. These literature data are consistent with our findings.

## Conclusion

In conclusion, biochemical, macroscopic, and histopathological analyses showed that cisplatin caused oxidative and inflammatory damage in the tongue tissue, reflecting mucositis. Additionally, these analyses revealed that ATP alleviated the oxidative and inflammatory damage caused by cisplatin in tongue tissue. Our results provide significant evidence regarding the protective and therapeutic efficacy of ATP in cisplatin-induced oral mucositis. These findings may contribute to the development of new therapeutic approaches to prevent or treat mucositis, a side effect so severe that can lead to treatment discontinuation. Moreover, we hope that our study will serve as a reference for future research and guide clinicians in developing more effective strategies for clinical practice.

## Data Availability

All data generated or analyzed during this study are included in this published article
